# Dental Treatment in Special Needs Patients and Uncooperative Young Children: A Retrospective Study

**DOI:** 10.3390/medicina60010091

**Published:** 2024-01-03

**Authors:** Matteo Biasotto, Augusto Poropat, Davide Porrelli, Giulia Ottaviani, Katia Rupel, Magdalena Theodora Bogdan Preda, Roberto Di Lenarda, Margherita Gobbo

**Affiliations:** 1 Department of Medicine, Surgery and Health Sciences, University of Trieste, 34125 Trieste, Italy; m.biasotto@fmc.units.it (M.B.); krupel@units.it (K.R.); theodora.bogdanc@gmail.com (M.T.B.P.); rdilenarda@units.it (R.D.L.); 2 Department of Life Sciences, University of Trieste, 34125 Trieste, Italy; dporrelli@units.it; 3 Unit of Oral and Maxillo Facial Surgery, Ca’ Foncello Hospital, 31100 Treviso, Italy

**Keywords:** Special Needs Patients, dental treatment, deep sedation, general anesthesia, special care in dentistry

## Abstract

*Background and Objectives:* Special Needs Patients (SNPs) and young non-collaborative children are more predisposed to develop oral pathologies due to poor collaboration and scarce access to dental treatment. The aim of this retrospective study was to analyze a sample of SNPs who received dental treatments either under general anesthesia (GA) or deep sedation (DS) over a period of 6 years. The number and type of procedure were analyzed. *Materials and Methods:* In total, 131 patients were included and mostly (>90%) treated under GA. Patients were either uncooperative and phobic (Group 1) or affected by mental, behavioral, and neurological disorders (Group 2), diseases of the nervous system (Group 3), or developmental anomalies (Group 4). *Results:* Patients in Group 2 required more invasive dental treatments than those in the other groups. Therapies were mainly preventive and restorative, except in Groups 3 and 4, where extractions were more frequent. The type of dental treatment significantly varied according to age and systemic condition. Only 5.3% of the patients needed a second intervention, despite only 17.6% of patients respecting the scheduled follow-up. *Conclusions:* Treatment under GA is effective, but the poor adherence to follow-ups and the risk of reintervention should be contrasted by improving the perception by parents/guardians of the importance of oral hygiene and periodic visits.

## 1. Introduction

The term “Special Need Patients” (SNPs) refers to people affected by health-related issues such as physical, developmental, mental, sensory, behavioral, cognitive, or emotional impairment or limiting conditions that require medical management, health care intervention, and/or the use of specialized services [1].

The incidence of oral pathologies in SNPs is high, both for poor or absent cooperation during home oral care and outpatient dental treatments, and for interactions between systemic pathologies, drugs, and oral health [2].

Historically, reluctance toward treating SNP was common since the promotion of oral health was considered less important than focusing on the main systemic pathology. Moreover, dental care was only provided on a complaint-based basis. Regular checkups during symptom-free intervals and routine professional tooth cleaning were not pursued [3]. This so-called “halo effect” was supported both by parents/guardians and by the medical team, which was frequently consulted only in emergency-urgency situations (pain, swelling, trauma) [4]. Furtherly hindered by the scarce cooperation of patients, treatments were basically conducted under general anesthesia (GA) or deep sedation (DS) in the operating room (OR), with higher biological (for the patients) and economic (for the healthcare system) costs providing mainly extractions, with scarce attention to prevention and teeth maintenance, even in young patients A similar behavior was applied to uncooperative young children. [5] While acknowledging the historical reliance on GA, it is imperative to recognize that, despite evolving perspectives that position it as a last resort, there are instances where a patient’s condition necessitates its use. In such cases, a proactive approach to prevention becomes paramount.

In recent years, increased awareness of the role of oral health as part of general wellness and life quality has fostered a profound change in the management of SNPs, further supported by awareness campaigns, family associations, and a sense of community. As a consequence, primary and secondary prevention have progressively gained a central role in the management of SNPs, reducing the need for DS and GA [6]. However, it is essential to acknowledge that despite the substantial decrease in the necessity for treatment in the OR in treating these patients, it remains a crucial modality for certain cases. Therefore, the literature must continue to gather comprehensive data to refine and optimize the management of patients for whom general anesthesia remains a necessary treatment modality.

Currently, there are few studies focused on dental treatments in uncooperative SNPs treated in the OR. The purpose of this retrospective study was to analyze a sample of SNPs treated in the OR to investigate the relationship between age and general health status and the type of dental treatments needed, type of intervention performed, type of anesthesia, necessity of second interventions, and adherence to scheduled recalls. In the same cohort, young uncooperative children were included, since they depend on parents/guardians at home and share common difficulties with SNPs during dental treatment.

## 2. Materials and Methods

SNPs and young uncooperative children were treated between January 2017 and December 2022 by the staff of the Oral Medicine and Pathology Unit (Maggiore Hospital, Trieste, Italy) in collaboration with the Department of Oral and Maxillofacial Surgery (Santa Maria degli Angeli Hospital, Pordenone, Italy) and with the Dental Clinic of Sacile Hospital. Patients were visited at the outpatient department either in Pordenone or in Sacile and operated under DP or GA in Pordenone. The data were retrospectively collected in 2023, and all the steps from the first visit, to the treatment choice and performing the treatment, to the follow up, as usually performed in our clinical setting, are described below. 

The first visit was performed by an associate professor in oral medicine, together with an anesthesiologist, at times aided by one collaborator (at least a 5-year post-graduate), and with the presence of parents and/or tutors of the patients. After a detailed collection of medical and dental histories, the patients underwent an intra- and extra-oral examination using behavioral management techniques (tell–show–do, behavioral desensitization) [7]. If the patients were collaborative, a panoramic dental X-ray was performed. Whenever feasible, attempts were made to perform treatments in the outpatients with behavioral techniques. Non-cooperative subjects were operated on with DS or GA. Parents and/or tutors were informed about the provisional treatment, which could be subdued to intra-operative changes due to objective difficulties in performing an accurate pre-operative examination. The level of cooperation of patients during the first visit and the American Society of Anesthesiologists Classification (ASA) risk were crucial to choosing the type of anesthesia [8]. When feasible, DS was preferred to GA, although at any moment, DS could be converted into GA.

The procedures performed on both deciduous and permanent teeth prior to local anesthesia included: oral hygiene and sealant application (preventive treatments), restorations (restorative treatments), root canal treatments (endodontic treatments), and extractions (surgical treatments). Maintenance of teeth was chosen whenever possible, although the treatment plan was customized on the basis of age, expected collaboration, and risk of relapse in the post-operative period. Silver amalgam was used only when a rubber dam could not be applied; composite resin restorations were preferred whenever dam isolation was feasible. Root canals were performed using mechanical instrumentation and single gutta–percha cone closure. In deciduous dentition, root canals were performed in teeth far from natural exfoliation and only when the risk of complications (i.e., onset of abscess or fistula) was low. In patients with mixed dentition, where root resorption was more advanced, extraction was usually preferred. 

Patients were usually dismissed after at least six hours after the end of the GA. At the end of the intervention, the parents and/or tutors were instructed on the correct techniques for home oral care and motivated to contact the hospital for a follow-up recall after 8 months.

Data regarding gender, age, systemic diseases, type of sedation, and the duration of intervention were collected. Medically compromised patients were recorded with a single code according to the International Classification of Diseases (ICD-11) [9]; the most severe and disabling diagnosis of every patient was decisive for the classification. Apart from uncooperative/dental-phobic patients, all the others fell into ICD-11 codes 6, 8, and 20. (Table 1). 

This study was conducted according to the guidelines of the Declaration of Helsinki and was approved by the Ethics Committee of the University of Trieste (number 113_2021). Informed consent was obtained from all subjects involved in this study.

### Statistical Analysis

OriginPro 2023 (OriginLab Corporation, Northampton, MA, USA) and Microsoft Excel 2019 software were used. Data that respected the normality of distributions are reported as mean and standard deviation; data that did not are reported as box plots. Occurrences are reported as histograms. For the statistical analysis, SNPs were divided into five different age ranges: less than 6 years (representative of primary dentition), between 7 and 12 years (representative of mixed dentition), between 13 and 25 years, between 26 and 45 years, and more than 46 years.

Data concerning the number of treatments per group, the age distribution in relation to the group, the duration of intervention in relation to type of anesthesia, and the age distribution according to the type of anesthesia, were analyzed with Kolmogorov–Smirnov’s and Levene’s tests to verify the normality of distributions and the homoscedasticity of the variances, respectively.

The number of treatments in relation to the pathology groups was analyzed with an ANOVA test and Tukey’s post hoc test.

The intervention time, age distribution, and mean number of treatments in relation to anesthesia type were analyzed with the Mann–Whitney U test for pairwise comparisons. Similarly, the age distribution in relation to the groups and the mean number of treatments in relation to the age groups were analyzed with a Kruskal–Wallis test and then a Mann–Whitney U test for pairwise comparisons with Bonferroni’s correction. χ^2^ tests were used for the following comparisons: type of sedation between the pathology groups, distributions of the ASA risk among the groups, distributions of the pathology in the age ranges, distributions of types of treatment in relation to the pathology group and age range, and the follow-up rate according to the pathology groups. The tests were performed by applying Bonferroni’s correction in cases of multiple-pair comparisons.

In all the analyses, the threshold for statistical significance was set at α = 0.05. 

## 3. Results

**Figure 1:**
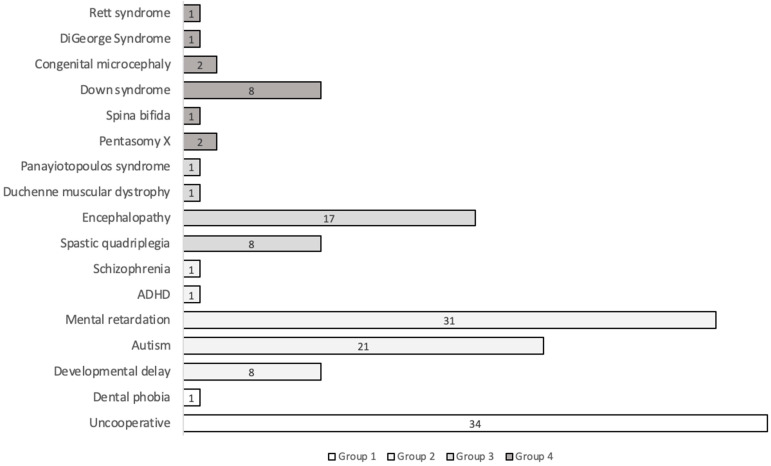
Distribution of SNPs according to pathology groups (ADHD: attention deficit hyperactivity disorder, presentation unspecified). Group 1: uncooperative and patients with dental phobia, Group 2: mental, behavioral, and neurological disorders, Group 3: diseases of the nervous system, and Group 4: developmental anomalies.

A total of 131 patients (69 males and 62 females) were treated between January 2017 and December 2022 (Figure 1). 

**Figure 2:**
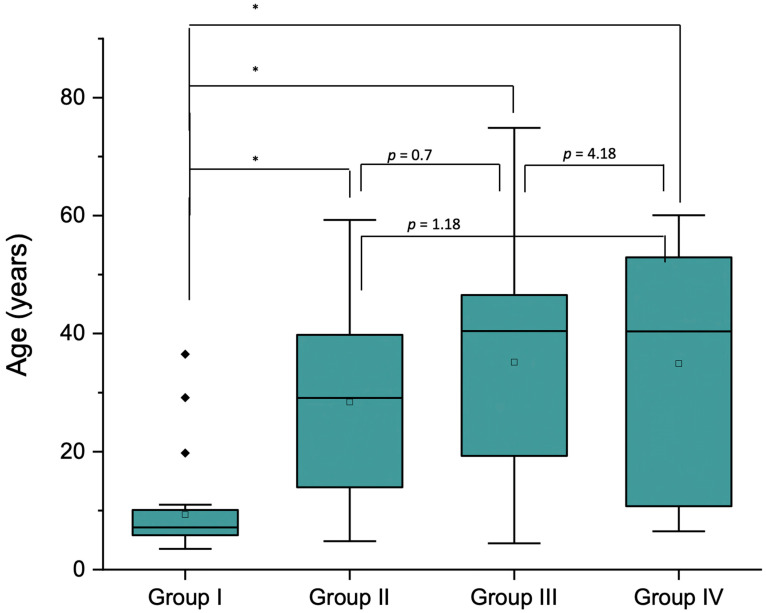
Box plot of the age (years) distribution for the four groups analyzed. Group 1: uncooperative and patients with dental phobia, Group 2: mental, behavioral, and neurological disorders, Group 3: diseases of the nervous system, and Group 4: developmental anomalies. * *p* < 0.001.

The mean age of patients was 25.3 ± 17.5 years (range 4.1–74.9). A total of 13 patients were younger than 6 years, 35 patients were between 7 and 12 years, 21 were between 13 and 25, 39 were between 26 and 45, and 23 were 46 years and older. Patients in Group 1 were significantly younger, whereas no statistically significant differences were found in the age distribution in the other groups. (Figure 2).

- **Significantly different**

**Figure 3:**
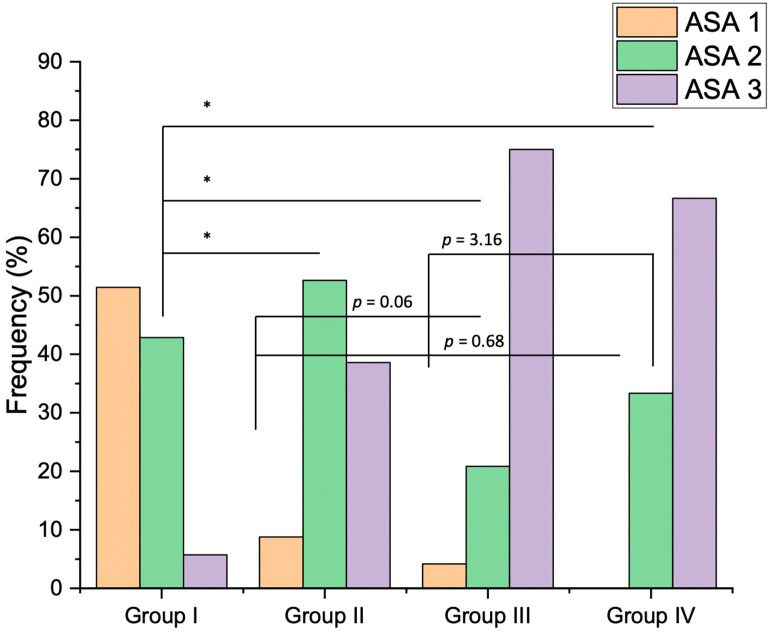
Distributions of ASA risk across study groups expressed as frequency (%). Group 1: uncooperative and patients with dental phobia, Group 2: mental, behavioral, and neurological disorders, Group 3: diseases of the nervous system, and Group 4: developmental anomalies. * *p* < 0.001.

Based on the ASA risk, 55 patients were classified as grade 2, 52 as grade 3, and 24 as grade 1, with significant difference in Group 3 (*p* < 0.001) (Figure 3).

**Figure 4:**
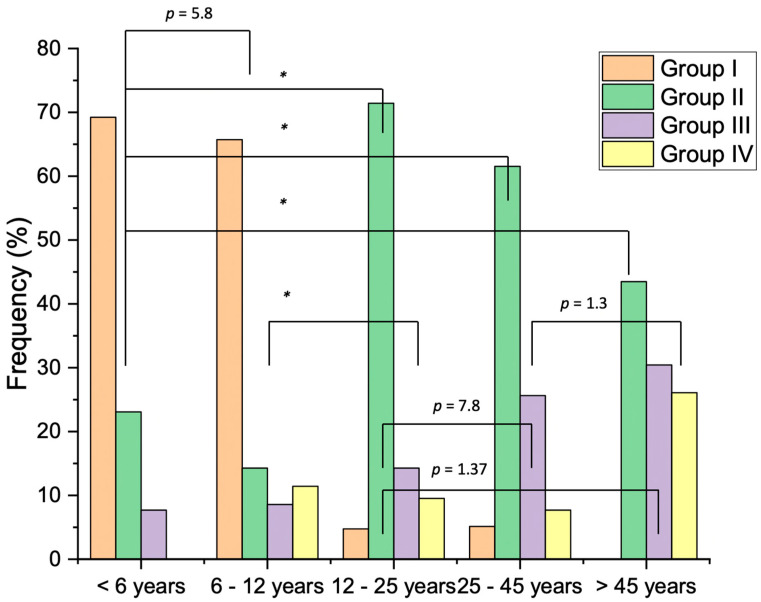
Distribution of conditions across various age ranges expressed as frequency (%). Group 1: uncooperative and patients with dental phobia, Group 2: mental, behavioral, and neurological disorders, Group 3: diseases of the nervous system, and Group 4: developmental anomalies. * *p* < 0.001.

Regarding the distribution of systemic diseases within the age groups, the differences were statistically significant (*p* < 0.001) between all the age ranges except for the comparisons including less than 6 vs. 7–12, 13–25 vs. 26–45, 13–25 vs. more than 46, and 26–45 vs. more than 46 years (Figure 4).

A total of 93% of patients underwent dental surgery in GA. A total of 3% of patients in Group 1, 11% in Group 2, and 8% in Group 3 were treated under DS; all interventions for Group 4 patients were performed under GA. However, no statistically significant differences in the prevalence of GA versus DS were found in relation neither to the pathology group nor to the type of sedation performed. A significantly lower average number of therapies was performed in patients treated with DS (2.7 ± 1.2) when compared with patients treated with GA (8.1 ± 5.1).

The overall mean duration of the intervention was 80.2 ± 44 min with a significant difference (*p* < 0.001) between the mean duration of dental treatments performed with DS (35 ± 22 min) versus GA (84 ± 44 min).

The mean number of dental treatments performed per patient was 7.7 ± 5.1 (range 1–25); restorative treatments were the most frequent (2.3 ± 1.6 on permanent dentition and 1.1 ± 1.2 on primary dentition), followed by extractive procedures (1.9 ± 1.7 on permanent dentition and 1.1 ± 1 on primary dentition) and root canal treatments (0.2 ± 0.3 both on permanent and primary dentition (Table 2). Overall, 83% of the preventive procedures were oral hygiene sessions.

**Figure 5:**
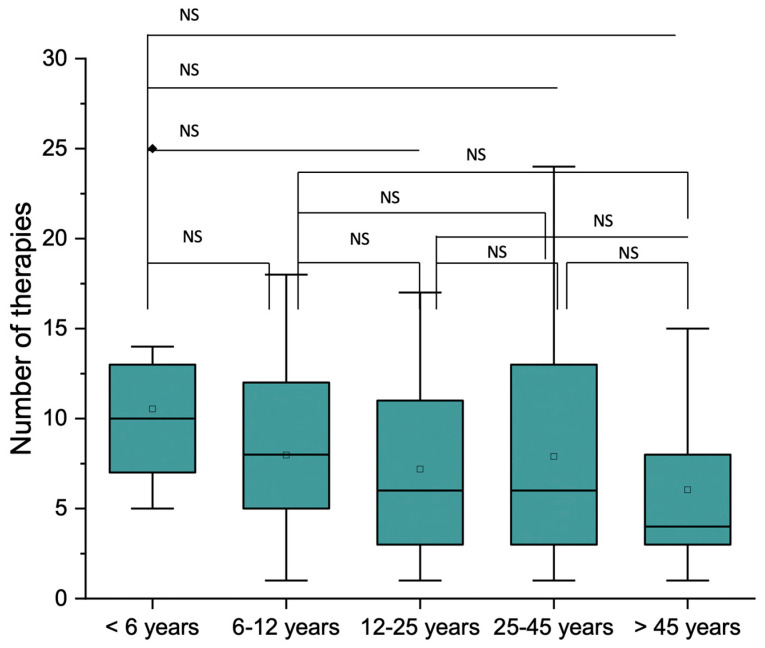
The mean number of treatments according to age ranges. No statistically significant differences in the mean number of treatments per patient in relation to the pathology groups or age ranges were found. NS: non-significant.

**Figure 6:**
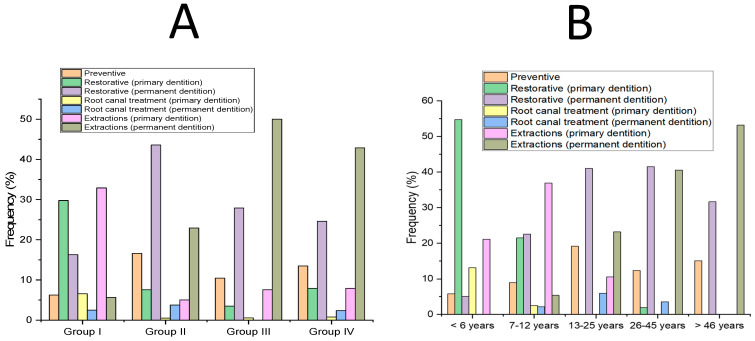
Frequency (%) of treatments per patient within pathology groups (**A**) and the frequency (%) of treatments per patient within age ranges (**B**). Group 1: uncooperative and patients with dental phobia, Group 2: mental, behavioral, and neurological disorders, Group 3: diseases of the nervous system, and Group 4: developmental anomalies. The differences in treatment distributions across various age intervals were statistically significant among all analyzed groups.

There were no statistically significant differences in the mean number of treatments per patient in relation to the pathology groups or age ranges (Figure 5), but the frequency of the type of therapies and treatment distributions were statistically significant (*p* < 0.001) between the groups (Figure 6). The frequency of performed therapies is summarized in Table 3. Only seven patients (5.3%) needed a second intervention with a mean time interval of 23.7 ± 13.4 months (range 5–45 months) (Table 4). 

ID_4 patient, affected by gingival hyperplasia related to anticonvulsant therapy for encephalopathy, underwent two scheduled maxillary gingivoplasty interventions within a short period. ID_2 patient, affected by severe mental retardation, was the only one treated three times with 7 restorations, reintervention on previously treated teeth.

Regarding the 8-month follow-up recall, only 17.6% of SNPs contacted the hospital without statistically significant differences between the four groups of pathologies.

## 4. Discussion

The use of GA to deliver dental care is often the only way for SNPs. While the success of DS also depends on a patient’s ability to cooperate, GA provides more safety for challenging patients because of airway management [10]. Furthermore, GA is associated with an increased risk of morbidity and mortality, so this modality should be chosen only when more conservative strategies are not feasible [11]. 

Moreover, the process of GA can be distressing for both patients and their families or care teams, and its use needs to be carefully considered. When chosen, the nature of care delivered may have to be modified, and guidance on how or when modifications to care should be made to support each individual is complex and lacks detailed guidance [12]. The main reason why, despite the potential anaesthesiological risks, this modality is still widely utilized, is that the final aim is to obtain a satisfactory life quality for patients.

Our results suggest that the pathological condition of the patients reflects different therapeutic needs. In fact, despite Groups 2, 3, and 4 being homogeneous in terms of age and ASA risk, patients underwent very different therapies (i.e., high frequency of extraction therapies in Groups 3 and 4). For this reason, we may infer that, despite including patients of very different ages, the choice of treatment is mainly related to the systemic pathological condition of patients.

The literature reports that dentists used to extract the teeth of SNPs under GA in the mistaken belief that this would avoid the need for further interventions. McGeown et al. refuted this hypothesis and strongly recommended evidence-based caries risk assessment and prevention as the main goal of SNP treatment [13]. We must not forget that the mouth represents a “calling card”, a first interface, so it is a fundamental element for the general well-being of individuals and acceptance by society [14]. For this reason, restorative treatment was performed whenever feasible and safe, especially for the frontal teeth. Nonetheless, especially for patients who did not feed orally, extractive therapies were preferred with the aim of minimizing the infections.

Differently from the present analysis, most of the studies investigating dental SNP treatments under GA include pediatrics [11], while only a few also include adults [15,16,17,18,19]. Our prevalence of uncooperative patients (young children and patients with seriously compromised oral status) with good general health and lower ASA risk was higher (27%) than other studies. In our study, Group 2 was the largest, consistent with Mallineni et al. [16], which had more than 60% of patients affected by the same conditions. On the contrary, Rothmaier et al. [20] found that 44.8% of SNPs were affected by congenital and chromosomal malformations, followed by patients with mental and behavioral disorders (13.8%). In the 7–12 years age group, differently from other authors who waited for natural exfoliation [21], we often decided to extract carious deciduous teeth close to natural exfoliation in order to prevent pain or annoyances during the post-operative period. 

Regarding the high frequency of permanent tooth extraction in the age groups 26–45 and over, we found a higher incidence of carious and/or periodontal lesions in older SNPs. A retrospective study on 4732 adult SNPs confirmed our findings [2] by reporting that the prevalence of untreated caries increases with age. This reflects the idea that the awareness of good oral health for promoting good QoL is a quite recent conquest. 

Chang et al. [22] evaluated the survival rate of teeth with single-visit endodontic treatment under GA in SNPs. The 5-year survival rate was significantly reduced in patients over 40 years (96.4 for SNPs younger than 40 years and 46.9 for SNPs over 40 years) and non-parental caregivers (96.2 for SNPs with a parental caregiver and 56.8 for SNPs with a non-parental caregiver); the poor cooperation level was also another risk factor. Mallineni et al. [16] reported a failure rate of pulp therapies at the end of a 24-month follow-up of 9.2%, with a mean age of the patients at the time of dental treatments of 12.3 ± 10.5 years. Other authors [23] preferred extractions to root canals in adults with the aim of avoiding the risk of complications and reinterventions. We believe that the choice should be personalized, considering general and dental conditions, in order to maintain teeth with a good endodontic prognosis without exposing the patients to the risk of pain.

Only a few studies in the literature analyzed the rate of repeated dental treatments in patients who underwent dental therapies under GA [24,25,26,27,28]; all of them focused on a pediatric population. Bücher et al. [25] focused on 10.8% of patients who underwent a second intervention, with a mean time elapsed between the two interventions of 28.9 ± 13.9 months. In our study, we had both a lower incidence of repeated intervention (5.3%) and average time between the two interventions (approximately 22.4 ± 13.9 months). In our study, only one patient needed to be treated for a third time; the rate of incidence was the same as in other studies [22,25,26,27,28]. Li JY et al. [29] showed that restoration failure was the main reason for a high unplanned retreatment rate, and the authors proposed strategies for a better outcome of GA like improving the professional knowledge and skills of operators and enhancing compliance of parents/patients. In our opinion, the limited time available in the OR and the need for treating a lot of teeth simultaneously may be considered a risk factor for reduced quality of the restorations. Thus, interventions should be followed by correct maintenance at home.

Still, too often, patients who undergo dental rehabilitation under GA do not return to the dentist until the pain returns, and further treatment under GA is needed. Thus, a vicious circle is created, and the symptomatic treatment under GA often means the loss of the causative tooth. Breaking this vicious circle requires, among other things, the expertise and implementation of behavioral guidance and communication techniques in the dental treatment of SNPs [30].

The awareness of the importance of good oral health as a reflection of good general status should be transmitted to parents and/or tutors, who must be informed and motivated to keep the situation under control: first, to limit the risk of discomfort, and second, to avoid further sessions in the OR. In these cases, prevention and oral hygiene instructions should be delivered at every appointment, including motivation regarding food intake and teeth washing.

The follow-up recall represents a key element. Unfortunately, almost all patients were not able to refer satisfaction or disappointment or the onset of pain in a specific area of the mouth or tooth due to their medical condition or their young age. Moreover, often, the treatment choices were based on the experience of the operator since intraoral X-rays could not be performed in the OR and no pain/disease history of each tooth was obtainable. This represents a limitation of the GA approach and another reason why outpatient intervention should be preferred whenever feasible. Our results appear to be worse than those of other studies. Mallineni et al. reported [16] that patients’ attendance to follow-up appointments (at 2 weeks and 6–12–18–24 months) declined significantly from 96% at the first appointment to about 80% at 6 months and 70% at 12 months.

The group of uncooperative patients was the one who returned to follow-up less frequently (6.7%); this may in part be attributed to logistic reasons since these patients are frequently difficult to move and bring to the hospital. Uncooperative young children not affected by disabling systemic diseases, which were already previously followed by their dentist but who could not be treated in the outpatient clinic, were recommended to contact their care provider if there had been episodes of pain or infection. This may have reduced the motivation to contact the hospital for a follow-up. Moreover, over the months/years, there was probably an improvement in the communication skills of the patients, who became more receptive toward behavioral techniques and thus were able to be treated in the outpatient clinic. This may also have negatively impacted the percentage of attended follow-up; so, in this case, the significantly younger age of patients in Group 1 may have played a role [7].

Efforts should be made to sensitize parents and guardians on the importance of follow-up [31], maybe involving the general practitioners (GPs), scheduling regular phone calls, or planning recall at the end of the surgery. A high level of sensitivity, empathy, and motivation on the part of the dental team is also essential. The long-term goal of these confidence-building measures is also to create an individualized chairside treatment situation that will reduce the need for GA in the future [3,32].

## 5. Conclusions

Despite having treated a significant number of patients, given the diversity of the pathologies included, a larger sample could provide insights regarding specific conditions. The treatment of the oral cavity in these subjects is aimed at enhancing their overall well-being. This study provides valuable data regarding the patients who are most frequently treated under GA and their progress. It is important to note that while general anesthesia enables comprehensive dental treatment in these patients, it is not without risks and potential sequelae. These considerations should be part of the decision-making process when planning treatment under general anesthesia. Future studies could benefit from a more detailed exploration of these risks and the long-term outcomes of these patients. It would be beneficial for future studies to not only focus on the immediate outcomes of the dental therapies performed in the OR but also to include a comprehensive follow-up of these patients and their progress over time. This could be further enriched by cooperation with other medical specialists, including the GP, the nutritionist, and the dentist. All these additional data would provide a more holistic view of the entire diagnostic, therapeutic, and follow-up journey in SNPs.

## Figures and Tables

**Table 1:** Pathology groups according to ICD-11.

Study Group	ICD Category	Description
Group 1	/	Uncooperative and patients with dental phobia
Group 2	ICD-6	Mental, behavioral, and neurological disorders
Group 3	ICD-8	Diseases of the nervous system
Group 4	ICD-20	Developmental anomalies

**Table 2:** Treatment procedures performed in the OR. The number of procedures per patient is expressed as mean ± SD.

Dental Treatment	Number of Procedures	Mean Number of Procedures Per Patient
Preventive	121	1.2 ± 0.5
Restorative	445	3.4 ± 1.8
Root canal treatment	51	0.4 ± 0.4
Extractions	397	3.0 ± 1.8

**Table 3:** Dental treatments in SNPs divided according to age range and pathology group. Data are presented as number of treated teeth per patient (%). The differences according to the pathology groups in every age range were always significantly different except in the age range of 26–45 between Groups 2 (mental, behavioral, and neurological disorders) and 3 (diseases of the nervous system) (exceptions are marked with *). Groups 2 and 4 (developmental anomalies) (exceptions are marked with **), and Groups 1 (uncooperative and patients with dental phobia) and 3 (exceptions are marked with ***). The differences according to age ranges within every pathology group were always significantly different except for 7–12 vs. 13–25 (exceptions are marked with §) and 13–25 vs. 26–45 years in Group 1 (exceptions are marked with §§); 13–25 vs. more than 46 years in Group 2 (exceptions are marked with §§§); less than 6 vs. 7–12, 7–12 vs. 13–25, 13–25 vs. more than 46l and 26–45 vs. more than 46 years in Group 3 (exceptions are marked with °); and 13–25 vs. 26–45, 13–25 vs. more than 46, and 26–45 vs. more than 46 years in Group 4 (exceptions are marked with °°).

Age	Group	Number of Patients		Primary Dentition			Permanent Dentition		
			Preventive	Restorative	Endodontic	Extraction	Restorative	Endodontic	Extraction
Less than 6 years	1	9	0.6 (4.8)	5.8 (49.5)	1.7 (14.3)	3 (25.7)	0.7 (5.7)	-	-
	2	3	1 (11.5)	6 (69.2)	0.7 (7.7)	0.7 (7.7)	0.3 (3.9)	-	-
	3	1	-	5 (83.3)	1 (16.7)	-	-	-	-
	4	-	-	-	-	-	-	-	-
7–12 years	1	23	0.6 (6.8)	1.9 (22.4)	0.3 (3.1)	3.4 (40.6)	1.7 (20.3)	0.2 (2.6)	0.3 (4.2)
	2	5	0.6 (7.5)	1.2 (15)	-	1.6 (20)	3.4 (42.5)	0.2 (2.5)	1 (12.5)
	3	3	0.7 (16.7) °	0.3 (8.3) °	-	2.3 (58.3) °	0.7 (16.7) °	-	-
	4	4	1.8 (20)	2.5 (28.6)	0.3 (2.8)	2.5 (28.6)	1.3 (14.3)	-	0.5 (5.7)
13–25 years	1	1	1 (33.3) §	-	-	-	1 (33.3) §	1 (33.3) §	-
	2	15	1.6 (21.0)	-	-	0.7 (8.8)	3.3 (43)	0.3 (4.4)	1.7 (22.8)
	3	3	0.7 (10.0) °	-	-	2 (30) °	2.3 (35) °	-	1.7 (25) °
	4	2	1 (14.3)	-	-	-	2.5 (35.7)	1.5 (21.4)	2 (28.6)
26–45 years	1	2	0.5 (5.3) ***§§	-	-	-	3 (31.6) ***§§	1 (10.5) ***§§	5 (52.6) ***§§
	2	24	1.1 (17.1) *	0.3 (3.8) *	-	-	3.5 (52.5) *	0.4 (5.7) *	1.4 (20.9) *
	3	10	0.7 (6.4) *	-	-	-	3.5 (31.8) *	-	6.8 (61.8) *
	4	3	1 (14.3) **°°	-	-	-	1.3 (19) **°°	-	4.7 (66.7) **°°
More than 46 years	1	-	-	-	-	-	-	-	-
	2	10	0.9 (15.2) §§§	-	-	-	2.3 (39) §§§	-	2.7 (45.8) §§§
	3	7	1 (29.1) °	-	-	-	0.6 (16.7) °	-	1.9 (54.2) °
	4	6	0.8 (8.9) °°	-	-	-	2.8 (30.4) °°	-	5.7 (60.7) °°

**Table 4:** List of SNPs who underwent a second intervention in the OR in the 2014–2019 period. Data on treatments are expressed as the number of therapies for every patient. Group 1: uncooperative and patients with dental phobia, Group 2: mental, behavioral, and neurological disorders, Group 3: diseases of the nervous system, and Group 4: developmental anomalies.

	Group	Age at Second Intervention (Years)	Time Interval (Months)	First Intervention				Second Intervention			
				Preventive	Restorative	Endodontic	Extraction	Preventive	Restorative	Endodontic	Extraction
ID_1	4	23,2	45	1	4	3	-	1	1	0	4
ID_2	2	25,5	36	1	6	1	0	1	3	-	-
ID_3	1	32,8	13	1	2	-	1	1	10	2	-
ID_4	3	15,8	5	1	-	-	5	1	1	-	2
ID_5	2	14,0	23	1	10	5	1	1	8	-	2
ID_6	1	10,5	18	1	3	1	-	1	3	-	1
ID_7	2	32,7	17	1	17	-	1	1	6	-	1

## Data Availability

Data are contained within the article.
